# Defining Loci in Restriction-Based Reduced Representation Genomic Data from Nonmodel Species: Sources of Bias and Diagnostics for Optimal Clustering

**DOI:** 10.1155/2014/675158

**Published:** 2014-06-25

**Authors:** Daniel C. Ilut, Marie L. Nydam, Matthew P. Hare

**Affiliations:** ^1^Department of Plant Breeding and Genetics, Cornell University, Ithaca, NY 14850, USA; ^2^Division of Science and Mathematics, Centre College, Danville, KY 40422, USA; ^3^Department of Natural Resources, Cornell University, Ithaca, NY 14850, USA

## Abstract

Next generation sequencing holds great promise for applications of phylogeography, landscape genetics, and population genomics in wild populations of nonmodel species, but the robustness of inferences hinges on careful experimental design and effective bioinformatic removal of predictable artifacts. Addressing this issue, we use published genomes from a tunicate, stickleback, and soybean to illustrate the potential for bioinformatic artifacts and introduce a protocol to minimize two sources of error expected from similarity-based de-novo clustering of stacked reads: the splitting of alleles into different clusters, which creates false homozygosity, and the grouping of paralogs into the same cluster, which creates false heterozygosity. We present an empirical application focused on* Ciona savignyi*, a tunicate with very high SNP heterozygosity (~0.05), because high diversity challenges the computational efficiency of most existing nonmodel pipelines while also potentially exacerbating paralog artifacts. The simulated and empirical data illustrate the advantages of using higher sequence difference clustering thresholds than is typical and demonstrate the utility of our protocol for efficiently identifying an optimum threshold from data without prior knowledge of heterozygosity. The empirical* Ciona savignyi* data also highlight null alleles as a potentially large source of false homozygosity in restriction-based reduced representation genomic data.

## 1. Introduction

As population genomic applications of next generation sequencing continue to expand beyond model organisms to nonmodel species and wild populations, reduced representation libraries (RRL) have been used to subsample genomes in a repeatable manner. Here, “nonmodel” refers to species with few genomic resources and in particular the absence of a reference genome. Early efforts, especially within plants, focused on RRL methods that could avoid highly repetitive genomic fractions. Two generalizable approaches to RRL construction have been Cot analysis to isolate the low-copy fraction of a genome [1] and screening of restriction enzymes for those that yield the desired fragment size range, in some cases taking advantage of enzyme methyl sensitivity to target certain genomic regions [2, 3]. Compared with Cot analysis, restriction digestion is a rapid and easy method of generating an RRL whether size selection is gel-based (e.g., RAD-tags, [4]) or a consequence of PCR and sequencing biases (genotyping-by-sequencing or GBS [5]). When a related genome sequence is available, in silico digests can help determine the optimum restriction enzyme with respect to fragment size distribution and avoid repetitive elements [6]. The simplicity of this restriction-based approach has made it the method of choice for genomic sampling apart from transcriptomes and hybridization-based sequence capture [7], but with the expectation that some portion of the data will be from multicopy DNA sequences and will possibly bias estimates of genetic diversity.

With nonmodel taxa, analysis of reduced representational data must contend with a potentially large fraction of repetitive DNA without the quality control benefits of mapping to a reference genome. Also, the clustering or binning of homologous sequences into allelic loci without the benefit of a reference genome has the potential to create multiple artifacts with different effects on inferred heterozygosity. When the goal is to simply find a set of single nucleotide polymorphisms to analyze spatially among population samples, stringent quality filtering can yield valuable data at the cost of enormous data loss. In contrast, when the goal is to identify individual candidate loci using genome scans or association mapping, or to make inferences requiring unbiased estimates of heterozygosity, results will be sensitive to biases and artifacts introduced by library construction, quality filtering, or allelic clustering methods. These latter applications are where the full and exciting potential of next generation sequence data hold the greatest promise to transform the questions that can be empirically addressed in natural populations, by making it possible to find and analyze both neutral and potentially nonneutral genomic variation. Therefore, our goal was to investigate the potential for large biases from repetitive DNA and copy number variation and explore novel de novo clustering methods that can minimize heterozygosity biases.

In what follows, we focus on reduced representation methods of genomic sampling such as RADseq and GBS. These methods focus sequencing effort on loci (sometimes referred to as “tags”) that are no larger than the sequence read length from high-throughput platforms (currently 100–150 bp read lengths). Usually in these protocols there is no “assembly” of partially overlapping sequences into longer contigs (e.g., [9]) but instead there is simple “stacking” or clustering of similar-sequence reads [10]. Our focus here is also on data from single individuals because many studies are barcoding individuals within multiplexed population samples, allowing for bioinformatic quality control and analysis at this informative organismal level when there is at least moderate (5–10 reads per locus) average coverage.

When clustering reads without a reference genome, a major biological factor affecting the potential for artifacts and biased heterozygosity is sequence diversity among paralogs versus alleles. First, the amount of repetitive DNA or large scale genomic duplication in the genome being sequenced is a potential concern, with high levels of recent duplication increasing the chances of mistakenly clustering paralogous sequences with alleles at a locus. However, typical measures of genome complexity and repetitive DNA content, when known, are of uncertain relevance for predicting the frequency of confounding paralogs at the specific scale of sequence read comparisons (~100 bp). Using high read counts to filter out problematic clusters is an intuitive approach to remove highly repetitive loci, but high stochastic variation in read counts among single copy loci makes it practical to remove only the most egregious and obvious artifacts with this type of filter. More recently a “ploidy informed” filter has been proposed wherein clustering involves all high-quality unique sequences but the resulting clusters with >2 distinct sequences per individual are removed from consideration [11, 12]. Because false heterozygosity can still result from clustering of two homozygous paralogous loci, particularly in more homozygous populations, one of our goals was to evaluate the effectiveness of the ploidy-based paralog filter.

**Figure 1:**
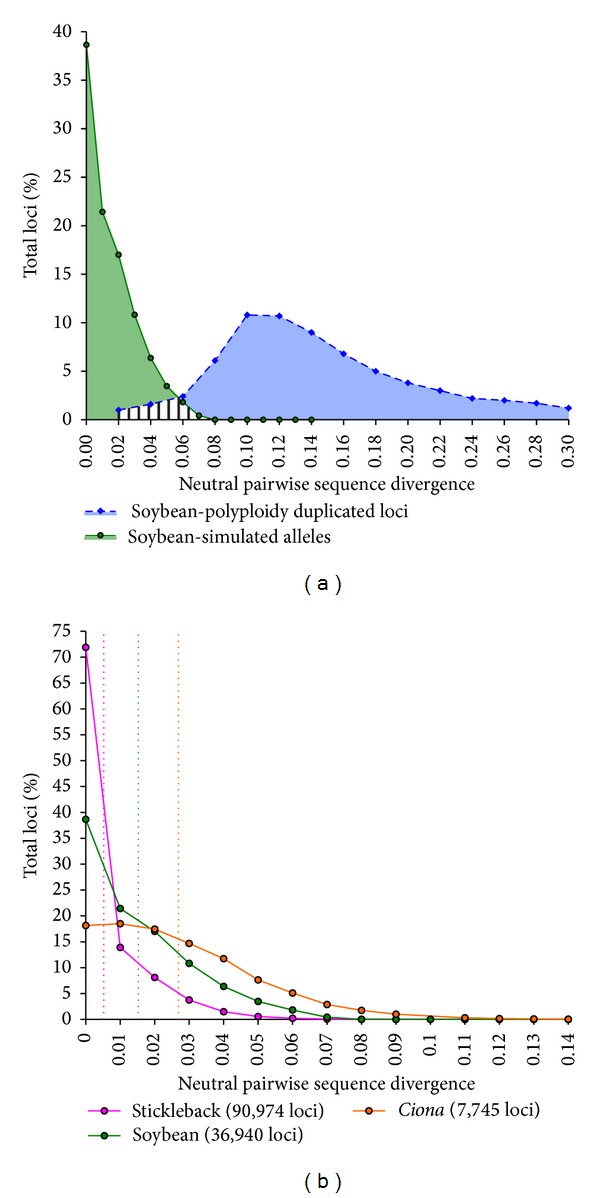
Pairwise divergence distribution for simulated allelic loci (green) and polyploidy duplicated loci (blue) in soybean (a), and pairwise divergence distribution of stickleback (pink), soybean (green), and* C. savignyi* (orange) simulated alleles (b). The shaded region in (a) between 0.02 and 0.08 pairwise divergence represents the “confounding duplication” region in which alleles and paralogs are indistinguishable during de novo assembly. The divergence of polyploidy duplicated paralogs in (a) is derived from [8]. Dashed vertical lines in (b) indicate distribution means. Different ranges are used for the *X*- and *Y*-*axis* values in (a) and (b).

Second, many natural populations have a wide range of sequence differences among selectively neutral alleles, and an even wider range must be considered to detect different forms of selection. In many cases, especially in populations with large effective population size and high heterozygosity or recent genome duplication events, the distribution of allelic sequence differences is likely to overlap with the distribution of paralogous sequence differences (Figure 1(a)), increasing the odds that paralogs will create false heterozygosity under a wide range of de novo clustering parameters. It is not possible to distinguish similarly divergent sequences from this distribution overlap without a reference genome, so either to be conservative or to be because of computational constraints, many studies and analysis pipelines consider only minimal allelic differences (1-2 bp per read; [10, 13]). Low allowable sequence differences will tend to split real alleles into separate clusters, or putative loci, creating false homozygosity. Other studies have used a subset of the data for computationally intensive de novo clustering allowing higher sequence difference thresholds, and after discarding clusters deemed to be artifact-prone used the concatenated contigs as a reference for mapping all sequence reads [11]. This latter procedure would benefit from an empirical method for determining, from the data, a minimum sequence difference threshold for clustering that eliminates false homozygosity. The optimum clustering threshold will strike a balance by minimizing false homozygosity, but avoiding higher thresholds because they increase computational demands and have greater potential for false heterozygous calls due to clustering of paralogs. Therefore, our second goal was to develop and assess a protocol for evaluating clustering thresholds using short read data in single individuals in order to avoid arbitrary thresholds and reduce both the amount of discarded data and the homozygosity bias of resulting data.

## 2. Methods and Materials

### 2.1. Simulations

#### 2.1.1. Genome Data

Genome assemblies were obtained for three species with published genomes: (1)* Gasterosteus aculeatus* (stickleback) genome assembly gasAcu1.0 ([14]; Broad Institute, http://www.broadinstitute.org/models/stickleback), (2)* Glycine max* (soybean) genome assembly Glyma1.1 ([8]; Joint Genome Institute, http://www.phytozome.net/dataUsagePolicy.php?org=Org_Gmax_v1.1), and (3)* Ciona savignyi* genome assembly version 2.1 ([15]; Broad Institute, http://mendel.stanford.edu/sidowlab/ciona.html).

#### 2.1.2. Genome Repetitiveness Evaluation

For each of the three genome sequences we defined a confounding divergence parameter (CDP) as the amount of divergence between paralogous sequences that would mirror average allelic divergence within the organism. For CDP in each species we used integer values approximating their genomic SNP heterozygosity: 1%, 2%, and 5% for stickleback, soybean, and* Ciona*, respectively [8, 15, 16]. Genome repetitiveness was estimated for each of the three genomes with the corresponding CDP value as follows: using the gem_mappability component of the GEM software library [17], each possible 100 bp fragment in the genome (sliding window, single bp increments) was assigned a value corresponding to the number of times that fragment had a match in the same genome with a divergence less than or equal to the CDP value for that genome. Single copy loci were identified as the class of 100 bp fragments with only one match.

#### 2.1.3. Read Simulation

For the three representative taxa we simulated the equivalent of paired-end Illumina GBS reads from single diploid genomes. We selected the six-base pair sequence GCAGCA as a digest pattern and performed in silico digest of the three genome assemblies using custom scripts. This GC-rich pattern was selected in order to preferentially select gene-rich regions [18, 19] that are typically targeted by the GBS protocol [5]. The sequence was selected to represent a general sampling of these regions, and does not correspond to any specific restriction enzyme. For* Ciona* the version 2.1 assembly used was a merger of the best segments from two haplome assemblies [15]. We removed any in silico digested fragments with a length greater than 800 bp or less than 200 bp, and read the first and last 100 bp of sequence from the remaining fragments.

From this pool of haploid sequences, we simulated an alternative allele for each locus in each species in a coalescent framework. First, we sampled a coalescence time for the two alleles from an exponential distribution with a rate of 1, followed by sampling the number of differences between the simulated alleles from a Poisson distribution with a rate equal to the coalescence time scaled by the average allelic divergence observed in the organism and the length of the sequence. Second, we calculated a per-base pair probability of mutation by dividing the number of differences sampled from the Poisson distribution by the length of the sequence. Finally, the 100 bp sequence (corresponding to a simulated error-free Illumina read) was duplicated and each nucleotide of the duplicate read was changed with probability equal to the per-base pair probability of mutation, replacing it with a nucleotide chosen with equal probability among the other three nucleotides. As a result of this process, for each genome we simulated allele pairs at each locus (referred to below as simulated reads) with a divergence between alleles sampled from a coalescence-adjusted Poisson distribution (Figure 1(b)). Indel polymorphisms and sequencing error were not simulated. A FASTQ [20] format file was generated for these reads, with the basecall quality value set to 40 for each nucleotide.

#### 2.1.4. Simulated Read Clustering and Clustering Threshold Optimization

The simulated reads for each genome were assembled de novo without the use of the reference sequence. First, the pool of reads was reduced to a nonredundant set using the tool SEED [21] allowing for no mismatches or gaps. Second, the nonredundant set was searched against itself for matching reads with a divergence of 15% or less using the tool SlideSort [22]. In order to illustrate the effects of splitting alleles into distinct clusters and combining paralogs into the same clusters, the 15% pairwise divergence threshold value for the high end of the clustering series was selected such that it was much larger than the largest average allelic pairwise divergence in our dataset (~5% in* Ciona*; [15]) and large enough to include polyploidy duplicated paralogs in soybean (Figure 1; [8]). Each read was paired to every other matching read sequentially, each pair of sequences and their divergence were recorded, and transitive clusters were built for all pairs whose divergence was below a given divergence threshold. A transitive cluster is a grouping of sequences such that, given three sequences A, B, and C, if the divergence between sequence A and B is below the chosen threshold, and the divergence between B and C is also below the threshold, then A, B, and C are grouped together regardless of the divergence between A and C. This clustering process was repeated for each integer threshold between 0 and 15%, and an “optimal threshold” was selected for each organism that maximized the number of clusters with two haplotypes and simultaneously minimized the number of clusters with one haplotype (see discussion). We selected clusters at the optimal mismatch threshold for further evaluation. Simulated data had no redundancy (multiple reads per allele), therefore read count thresholds were not applied and sequence sampling error was not evaluated.

#### 2.1.5. Simulated Clustering Evaluation

For the simulated data each haplotype had a known chromosomal origin, and each locus contained at most two haplotypes. Therefore, the heterozygosity of the simulated data can be computed directly, and the difference between the exact simulated heterozygosity and the inferred heterozygosity from de novo clustering represents the expected inaccuracy of assemblies for the given organism that results solely from the interaction between simulated allelic divergence, the amount of genome duplication, and the clustering threshold employed.

### 2.2. *Ciona savignyi* Sequencing and Clustering

#### 2.2.1. Source Material, Library Preparation, and Sequencing

Genomic DNA from a wild-collected single diploid* Ciona savignyi* individual, previously used for genomic sequencing [15], was kindly provided by A. Sidow. The DNA was prepared for genotyping-by-sequencing as described in Elshire et al. [5] and barcoded separately from other samples. Paired-end (2 × 88 nucleotides) data was collected on an Illumina Genome Analyzer (Illumina Inc., San Diego, California, USA) in the Cornell Laboratory Core.

#### 2.2.2. Sequence Cleanup and Clustering

Using custom scripts, raw sequence files in the QSEQ format [23] for an individual were scanned for any barcode tag, sequencing adaptor, and enzyme cut site sequence and these were trimmed from the sequence ends. After trimming we removed from analysis any sequences shorter than 75 bp, containing internal enzyme cut sites, or containing at least one nucleotide quality value less than 20. The remaining sequences were assembled into unique tag clusters using the same pipeline as the simulated reads (see above), with the following modifications: (1) any tag that did not have at least four confirming reads was discarded from further analysis prior to the nonredundant reduction step using SEED, and (2) in the SlideSort pairwise matching step, internal indel mismatches were counted as one mismatch regardless of indel size, and edge indels were not counted towards the total number of mismatches between two reads. Finally, to reduce the probability of false homozygosity due to sequence sampling error below 1%, given a minimum of 4 reads supporting each allele (calculations in Supplementary Material S1; see S1 in Supplementary Material available online at http://dx.doi.org/10.1155/2014/675158), we only analyzed clusters (putative loci) that had 11 or more reads assigned to them within the single individual.

## 3. Results

### 3.1. Genome Characteristics

**Figure 2:**
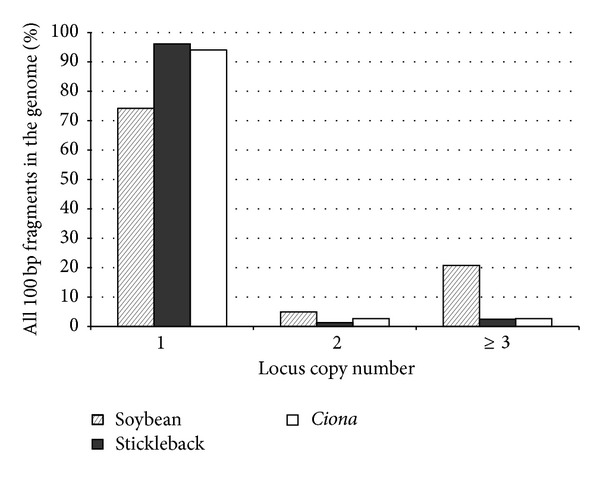
Potentially confounding duplication across all possible 100 bp fragments in three genomes. At this scale of comparison most loci are single-copy in each genome (copy class 1) and do not present a paralog clustering problem when trying to de novo assemble alleles. Paralogous loci from copy class 2 have the potential to be clustered together and if each paralog is homozygous, the cluster will be incorrectly interpreted as a diploid heterozygous locus even after a ploidy-informed filter. In principle, a ploidy-informed filter should be able to identify and remove most clusters derived from loci in the 3+ copy class.

The three genomes selected for study vary greatly in size, GC content, and amount of duplication within the genome. For stickleback, the reference genome sequence is 401 Mbp in size with a GC content of 43.49%. The soybean reference genome is 974 Mbp in size with a GC content of 34.10%, and the* C. savignyi* reference genome is 177 Mbp in size with a GC content of 35.89%. The amount of genome repetitiveness is presented in Figure 2, with the 1-copy class representing the proportion of 100 bp loci that had a single copy in the genome, the 2-copy class representing the proportion of loci that had exactly one other (paralogous) match in the genome, and the 3+ copy class representing the proportion of loci with multiple matches across the genome. As expected given its history of whole genome duplication [24], soybean had the largest percentage of loci duplicated across the genome. When sequencing reduced representation libraries, sequences sampled from the 2 and 3+ copy classes for a given organism have the potential to confound de novo clustering of reads as their paralogous divergence is within the expected divergence range for alleles within the organism (Figure 1(a)).

### 3.2. Simulated and Sequenced Reads

Summary of statistics for the simulated GBS data are presented in Table 1. For the experimental data from a* C. savignyi* individual, we obtained 12,188,018 high quality reads of 75 bp each. These reads were collapsed into 3,033,429 nonredundant sequences (representing both allelic variants and different loci). Of these, 427,919 (14.11%) had 4 or more reads supporting the tag sequence, and these were used for further analysis.

### 3.3. Clustering Threshold Series

**Figure 3:**
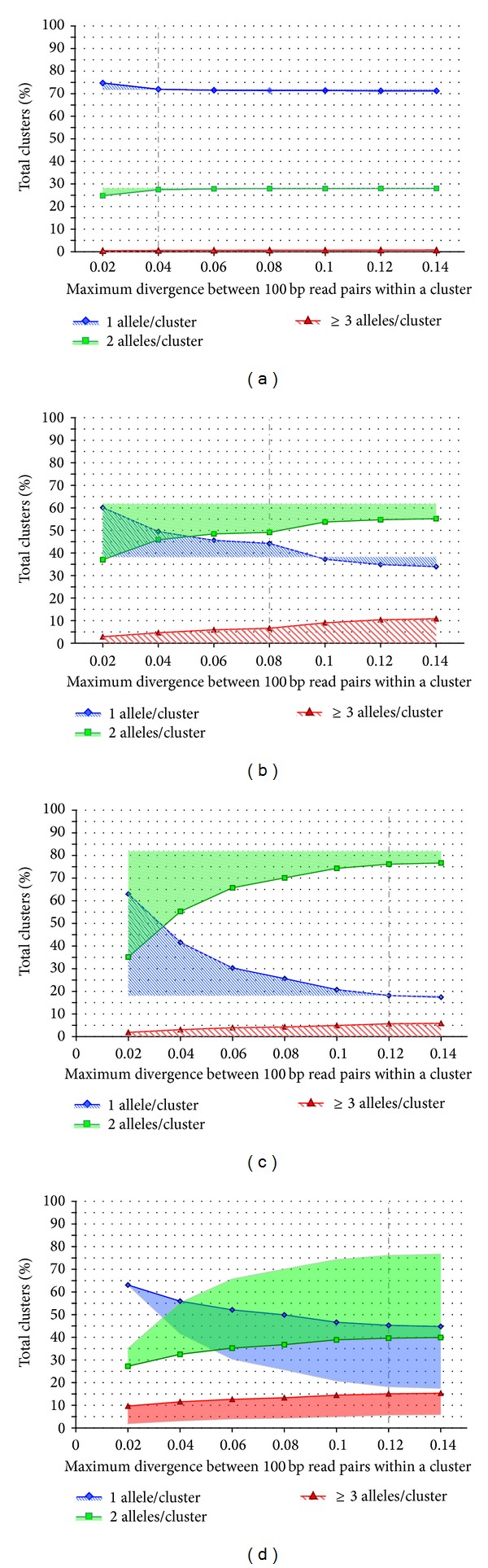
Clustering mismatch threshold series for simulated stickleback reads (a), simulated soybean reads (b), simulated* C. savignyi* reads (c), and experimental* C. savignyi* data (d). The *Y*-*axis* represents the percentage of total clusters for a given organism at a given mismatch value and the *X*-*axis* represents the maximum proportion of differences (mismatches) allowed between reads within a cluster. Single haplotype clusters (putative homozygous loci) are represented by a solid blue line and diamonds, two-haplotype clusters (putative heterozygous loci) are represented by a solid green line and squares, and three or more haplotype clusters (combined alleles from 2 or more paralogous loci) are represented by a solid red line and triangles. Striped shaded areas for simulated data represent deviation from the true values due to assembly artifacts such as splitting alleles into different clusters when the threshold is low or combining paralogs into a cluster when the threshold is high. Solid shaded areas for experimental data represent deviation from the expected simulated values due to assembly artifacts and null alleles. The uptick in heterozygosity observed between 0.8 and 0.10 in (b) is likely a result of the surge in 2+ paralog clustering over the same interval, perhaps due to clustering of duplicated loci from the soybean polyploidy event. The mean values of total cluster counts across assembly thresholds for plots (a)–(d) are 88300 (SD = 1987), 29201 (SD = 4798), 7700 (SD = 1244), and 167824 (SD = 10863).

For each of the three simulated data sets and the experimental* Ciona* data, we generated clustering threshold series in order to evaluate the impact of the maximum allowable mismatch threshold on the proportion of clusters free from artifacts. The simulated data for all three species showed a trend with increasing mismatch thresholds from 2% to 14% whereby single haplotype clusters (putative homozygous loci) decreased and two-haplotype clusters (putative heterozygous loci) increased (Figures 3(a)–3(c)). The greatest degree of disparity between simulated read cluster heterozygosity and true simulated heterozygosity occurred at low mismatch thresholds. For simulated stickleback reads, clusters with only one haplotype were minimized at mismatch thresholds of 4% and greater; at lower mismatch values oversplit alleles inflated the total cluster number and apparent homozygosity (Figure 3(a)). The number of clusters with 3 or more distinct sequences was negligible at all thresholds, and therefore the number of both homozygous and heterozygous clusters converged to the true value once the clustering threshold was raised to ≥4%. For simulated soybean (Figure 3(b)) the symmetry of changes in cluster proportions is more apparent as increasingly larger mismatch thresholds convert oversplit 1-haplotype clusters into heterozygous clusters. The magnitude of the artifact at low mismatch thresholds scales with heterozygosity. As mismatch thresholds increase, the inferred number of heterozygous clusters increases dramatically in both soybean (37% to 55%, Figure 3(b)) and* Ciona* (~35% to 77%, Figure 3(c)). The proportion of paralogous clusters slowly increases with increasing mismatch threshold but is typically small, reaching a maximum among these species of 11% in soybean at a 14% mismatch threshold. Because artifactual heterozygosity in the 2-haplotype class is expected to be minor, these results suggest that an optimum mismatch threshold would be the point at which there exists 1-haplotype and 2-haplotype cluster proportions asymptote or 4%, 8%, and 12% maximum sequence difference for stickleback, soybean, and* Ciona*, respectively (Figure 3, vertical dashed line). At higher mismatch thresholds there may be diminishing returns, with paralogs contributing to more new 2-haplotype clusters than collapsing oversplit clusters. The experimental* C. savignyi* data (Figure 3(d), sequence differences included variant nucleotides and indels) showed the same tendency to asymptote at higher sequence difference thresholds, but a large bias toward homozygosity remained at the asymptote.

### 3.4. Computational Streamlining of Threshold Determination

For experimental data sets from high heterozygosity species it can be computationally intensive to determine the cluster category distribution at large sequence difference thresholds. For the* C. savignyi* experimental data the calculation across the entire clustering threshold range took approximately 4 days on a modern quad-core Intel processor machine with 16 GB RAM, with the time limiting step being the SlideSort pairwise alignment. Two procedures were implemented to streamline these computations. First, clustering with a full all by all alignment was restricted to the maximum sequence difference threshold, and cluster results at lesser thresholds were rapidly estimated by pruning according to pairwise sequence difference. Specifically, we recorded the pairwise distance between all reads in the set used for maximum difference threshold clustering. Then to estimate cluster results for each lower threshold, read pairs with a divergence above this new threshold were separated as unpaired reads, and transitive clusters were built anew to account for multiread clusters being separated into new 1-read and 2-read clusters. Second, in order to evaluate whether subsampled data could generate robust clustering threshold series to determine an asymptote, we generated the maximum divergence clustering series (14 bp difference) using subsets of the* C. savignyi* experimental data uniformly subsampled without replacement to represent a range between 10% and 100% of the total reads. It should be noted that subsampling can inflate specific estimates of homozygosity due to unsampled alleles, and estimates of these specific values should be done with the full data set. However, as shown in Figure  S2 the shape of the clustering series curve generally holds at all levels of subsampling, and it quickly converges to the shape and values of the full data set once 20–30% or more of the data are used.

## 4. Discussion

In order to explore genomic repetitiveness and allelic divergence distributions with simulated data, we selected three reference genomes from organisms with very different genomic parameters. The two genomes with low amounts of duplication (stickleback and* Ciona*, Figure 2) have very low and very high heterozygosity, with 0.5% and ~5% average divergence between alleles in stickleback and* Ciona*, respectively. The soybean genome, by contrast, has a history of polyploidy leading to large parts of the genome being maintained in multiple copies (Figure 1) but its heterozygosity falls in between the two extremes above. Simulated data from these genomes is therefore expected to illustrate the effects of duplication and heterozygosity on clustering quality under different read clustering regimes. However, since additional factors contribute to clustering errors in an experimental dataset, we also compared the results of our simulations with de novo assemblies of reads from genotyping-by-sequencing results on the genome of the same individual* C. savignyi* specimen that was used to generate the publicly available reference genome sequence.

### 4.1. The Paralogs Problem

Regarding the issue of false heterozygous clusters due to the clustering of paralogs, our simulated data shows that even in the case of a highly duplicated genome their impact is relatively low (Figure 3(b)). Partly, this is because only duplications with divergences within the same range as allelic divergence present a problem for clustering. With the exception of extremely recent polyploidy events, the majority of maintained duplicated loci are expected to be much older (have much higher divergence) than the alleles in a population, resulting in only a small number of duplicated loci exhibiting “confounding divergence.” For example, duplicates from the most recent polyploidy event in soybean (~14 million years ago (mya); [25]) have a mean pairwise divergence of ~10–20% [8, 24, 25]. The age of the soybean polyploidy event is typical of most recent polyploidy events in other crop species such as maize (~12 mya; [26]) and cotton (~13–20 mya; [27]). Therefore, a ploidy-informed cut-off on the number of haplotypes per cluster per individual appears to be sufficient to address this issue in most cases. This result suggests that when devising clustering methods that minimize paralog clusters while also minimizing the splitting of divergent alleles into artifactual 1-haplotype clusters, the former problem is largely ameliorated by filtering out 3+ haplotype clusters. This provides flexibility to apply generous sequence difference thresholds to maximize allelic clusters without large loss of data from the elimination of putative paralog clusters. In our simulated soybean data, only ~7% of putative loci were eliminated by this filter when selecting the maximum 8 differences threshold (Figure 3(b)).

### 4.2. Insights from Simulated Data

Given the small amount of paralog-induced erroneous clustering observed, the clustering quality is mainly reflected by the change in relative proportions of 1-haplotype and 2-haplotype clusters over different sequence difference thresholds. As the clustering threshold increases up to the maximum divergence between alleles, oversplit allelic clusters are collapsed, decreasing the proportion of 1-haplotype clusters, with a concomitant increase in 2-haplotype clusters. Clustering thresholds higher than allelic divergence are not expected to alter observed counts of 1-haplotype and 2-haplotype clusters except for new clustering errors from paralogous loci. Indeed, as the simulated data in Figure 3 shows, cluster counts plateau once the threshold reaches a certain value: 4 bp difference for stickleback, 8 bp for soybean, and 12 bp for* Ciona*. Only soybean exhibits a secondary abrupt increase in heterozygous clusters at high clustering thresholds due to its polyploid nature. This suggests that as paralog clusters accumulate at higher mismatch thresholds in species that have not recently undergone whole genome duplication, they mostly fall into the 3+ read class and are easily filtered out. Very few 2-haplotype clusters are accounted for by clustering two homozygous paralogs. These simulated data comparisons suggest that the clustering threshold series asymptote marks an optimal clustering threshold regardless of genomic heterozygosity because at that point collapsing of alleles is fully realized but hidden paralog artifacts are typically minimal. Even for model species the distribution of allelic differences in a particular population will be unknown, so it is this empirical basis for identifying the optimum clustering threshold that makes this protocol valuable.

### 4.3. Evaluating Clustering Thresholds with Real Data

From a computational perspective, the main challenge an investigator faces when attempting de novo clustering in a high heterozygosity organism is the practical computational constraints on clustering with large sequence divergences allowed. Many tools such as Stacks [10] use a k-mer based approach to clustering, and its memory requirements increase exponentially with the average number of allowed mismatches between reads. Therefore, it is often impractical to attempt clustering with more than 2 or 3 mismatches allowed between reads, and in fact some tools limit this parameter to one mismatch (e.g. UNEAK [13]). As our simulated data show (Figure 3), low threshold clustering can easily misidentify heterozygous loci as homozygous by splitting alleles into different locus clusters even in species with very low heterozygosity. For example, a 2.5% difference in estimated heterozygosity is observed for stickleback when moving from 2% to 4% sequence difference thresholds (Figure 3(a)). However, while extremely useful in assembling partially overlapping reads at a locus, k-mer based approaches are not necessary (or ideal) for analyzing restriction based RAD-tag or GBS type data that produce sequence reads always starting at the same position for a given locus. Such data are better suited for a classic all-versus-all read comparison, which does not have as severe computational divergence limitation. We chose SlideSort [22] for our analysis, but other similar tools have been available for over a decade and have been used by other groups for similar purposes (e.g., CAP3, BLAST).

From a biological perspective, ideal clustering thresholds are highly species dependent and therefore a priori thresholds that worked well in one species might not work in another. Our clustering series approach allows an investigator to determine an optimal threshold for a given sample directly from the data. To our knowledge, this is the first time such an approach has been presented, and the very different optimal thresholds obtained for the three species simulated here suggest that such an analysis is worthwhile in order to improve the quality of de novo assemblies of restriction-based genome samples.

### 4.4. Null Alleles

An important question is whether the approach presented here will work as well to identify clustering mismatch thresholds in experimental data as it does with simulated data. At first glance the large difference between homozygosity asymptotes in simulated versus experimental* Ciona* data suggests caution in generalizing from the former. However, the observed discrepancy can easily be explained as an expected result of restriction-based sampling of a high heterozygosity species. Heterozygous restriction sites will generate sequence data for only one of the two homologous chromosomes. The frequency of loci with these null alleles is directly proportional to genomic heterozygosity. Impacts of this biased sampling include inflated estimates of homozygosity, increased coalescent variance across loci, and increased heterogeneity of locus sampling among individuals (for polymorphic restriction sites, the locus will not get sampled in homozygous null individuals). In population samples the bias can be effectively reduced by narrowing analyses to the set of loci represented in all individuals [28], but only at the cost of large data loss, so model-based filters are needed. Methods for estimating the proportion of unsampled loci due to heterozygous restriction sites have been previously developed [29], and based on these calculations, we expect at least 24% of the loci in* C. savignyi* to be falsely identified as homozygous due to variation at restriction sites resulting in null alleles. Homozygosity in* C. savignyi* simulated data, at the optimal clustering threshold and with no null alleles, is 18%. Therefore, the expected homozygosity in the experimental data when accounting for the expected null alleles is at least 42%, in agreement with the observed 45% from the experimental data.

## 5. Conclusions

In general, the clustering of paralogous sequences is a minor source of error in restriction-based reduced representation data for many species and can be effectively addressed by ploidy-based filtering. False homozygous loci, however, can produce a potentially strong bias, and both mechanisms potentially generating this bias, oversplit alleles and null alleles, have effects that scale with heterozygosity. We have provided formulae to calculate the read count filter necessary to achieve a given expected amount of false homozygous loci due to lack of second allele sampling during sequencing (Eq. iii, Supplementary 1). More importantly, we demonstrated an empirical procedure to determine a clustering mismatch threshold that minimizes the splitting of alleles into artifactual 1-haplotype clusters. The clustering threshold series approach can help select the appropriate cut-off for clustering the full data set without requiring any a-priori knowledge about heterozygosity in the organism under study, and this diagnostic step can be done with a subsample of the data in order to accelerate analysis.

## Supplementary Material

Suplementary materials present the derivation of a formula for allele sampling error expectation given a read redundancy threshold (S1), the effect of data sub-sampling on clustering series attributes (S2), and a flowchart schematic of the clustering series protocol (S3).

## Figures and Tables

**Table 1:** Simulated restriction-based genome sampling for the three species.

Species	Total fragments	200–800** **bp fragments	Total 100** **bp reads	Homozygous Tags percentage	Heterozygous Tags percentage	Mean allele divergence
Stickleback	345,704	90,974 (26.32%)	181,948	72%	28%	0.5%
Soybean	269,756	36,940 (13.69%)	73,880	39%	61%	1.5%
*Ciona *	61,102	7,746 (12.68%)	15,492	18%	82%	2.7%

## References

[1] Peterson DG, Schulze SR, Sciara EB (2002). Integration of cot analysis, DNA cloning, and high-throughput sequencing facilitates genome characterization and gene discovery. *Genome Research*.

[2] McCouch SR, Kochert G, Yu ZH (1988). Molecular mapping of rice chromosomes. *Theoretical and Applied Genetics*.

[3] Rabinowicz PD, Schutz K, Dedhia N (1999). Differential methylation of genes and retrotransposons facilitates shotgun sequencing of the maize genome. *Nature Genetics*.

[4] Baird NA, Etter PD, Atwood TS (2008). Rapid SNP discovery and genetic mapping using sequenced RAD markers. *PLoS ONE*.

[5] Elshire RJ, Glaubitz JC, Sun Q (2011). A robust, simple genotyping-by-sequencing (GBS) approach for high diversity species. *PLoS ONE*.

[6] van Tassell CP, Smith TPL, Matukumalli LK (2008). SNP discovery and allele frequency estimation by deep sequencing of reduced representation libraries. *Nature Methods*.

[7] Davey JW, Hohenlohe PA, Etter PD, Boone JQ, Catchen JM, Blaxter ML (2011). Genome-wide genetic marker discovery and genotyping using next-generation sequencing. *Nature Reviews Genetics*.

[8] Schmutz J, Cannon SB, Schlueter J (2010). Genome sequence of the palaeopolyploid soybean. *Nature*.

[9] Etter PD, Preston JL, Bassham S, Cresko WA, Johnson EA (2011). Local de novo assembly of rad paired-end contigs using short sequencing reads. *PLoS ONE*.

[10] Catchen JM, Amores A, Hohenlohe P, Cresko W, Postlethwait JH, de Koning D-J (2011). Stacks: building and genotyping loci de novo from short-read sequences. *G3, Genes, Genomes, Genetics*.

[11] Parchman TL, Gompert Z, Mudge J, Schilkey FD, Benkman CW, Buerkle CA (2012). Genome-wide association genetics of an adaptive trait in lodgepole pine. *Molecular Ecology*.

[12] Peterson BK, Weber JN, Kay EH, Fisher HS, Hoekstra HE (2012). Double digest RADseq: an inexpensive method for de novo SNP discovery and genotyping in model and non-model species. *PLoS ONE*.

[13] Lu F, Lipka AE, Glaubitz J (2013). Switchgrass genomic diversity, ploidy, and evolution: novel insights from a network-based SNP discovery protocol. *PLoS Genetics*.

[14] Jones FC, Grabherr MG, Chan YF (2012). The genomic basis of adaptive evolution in threespine sticklebacks. *Nature*.

[15] Small KS, Brudno M, Hill MM, Sidow A (2007). A haplome alignment and reference sequence of the highly polymorphic Ciona savignyi genome. *Genome Biology*.

[16] Hohenlohe PA, Amish SJ, Catchen JM, Allendorf FW, Luikart G (2011). Next-generation RAD sequencing identifies thousands of SNPs for assessing hybridization between rainbow and westslope cutthroat trout. *Molecular Ecology Resources*.

[17] Derrien T, Estellé J, Sola SM (2012). Fast computation and applications of genome mappability. *PLoS ONE*.

[18] Aïssani B, Bernardi G (1991). CpG islands, genes and isochores in the genomes of vertebrates. *Gene*.

[19] Sumner AT, de la Torre J, Stuppia L (1993). The distribution of genes on chromosomes: a cytological approach. *Journal of Molecular Evolution*.

[20] Cock PJA, Fields CJ, Goto N, Heuer ML, Rice PM (2009). The Sanger FASTQ file format for sequences with quality scores, and the Solexa/Illumina FASTQ variants. *Nucleic Acids Research*.

[21] Bao E, Jiang T, Kaloshian I, Girke T (2011). SEED: efficient clustering of next-generation sequences. *Bioinformatics*.

[22] Shimizu K, Tsuda K (2011). Slidesort: all pairs similarity search for short reads. *Bioinformatics*.

[23] Illumina Incorporation

[24] Shoemaker RC, Schlueter J, Doyle JJ (2006). Paleopolyploidy and gene duplication in soybean and other legumes. *Current Opinion in Plant Biology*.

[25] Schlueter JA, Dixon P, Granger C (2004). Mining EST databases to resolve evolutionary events in major crop species. *Genome*.

[26] Swigonová Z, Lai J, Ma J (2004). Close split of sorghum and maize genome progenitors. *Genome Research*.

[27] Wang K, Wang Z, Li F (2012). The draft genome of a diploid cotton Gossypium raimondii. *Nature Genetics*.

[28] Davey JW, Cezard T, Fuentes-Utrilla P, Eland C, Gharbi K, Blaxter ML (2013). Special features of RAD Sequencing data: implications for genotyping. *Molecular Ecology*.

[29] Upholt WB (1977). Estimation of DNA sequence divergence from comparison of restriction endonuclease digests. *Nucleic Acids Research*.

