# Supplementary material for: Defining Loci in Restriction-Based Reduced Representation Genomic Data from Nonmodel Species: Sources of Bias and Diagnostics for Optimal Clustering
Source: Biomed Res Int. 2014 Jun 25;2014:675158. doi: 10.1155/2014/675158 (PMC4095725; doi:10.1155/2014/675158)
Supplement: Supplementary file 1 — Suplementary materials present the derivation of a formula for allele sampling error expectation given a read redundancy threshold (S1), the effect of data sub-sampling on clustering series attributes (S2), and a flowchart schematic of the clustering series protocol (S3). [file 675158.f1.pdf]

S1. Read depth filtering

With individual-level barcoded data, the error rate for miscalling heterozygotes as homozygotes or vice versa depends both on the sampling process from the two homologous chromosomes and on the read coverage necessary to filter out sequencing error. These two error sources interact to require higher coverage. The probability of sampling only one chromosome from a heterozygous locus (false homozygote) is binomial. For example, with an average 8x coverage, among loci where that coverage is achieved there will be ~6.25% that yield one sequence from the first chromosome and seven sequences from the second chromosome, making sequencing error impossible to evaluate. If sequencing error is not being estimated from the data, rather (more typically) filtered out at each locus based on an arbitrary level of sequence redundancy, then >>1 read per locus is required to distinguish true, error-free sequences from those with error. Otherwise, sequencing errors will turn homozygous loci into false heterozygous loci, introducing bias, or heterozygous loci into apparent paralog clusters, causing bias or loss of data. For an arbitrary error-filtering coverage requirement, we sought to determine the coverage necessary to achieve a given probability of sampling both chromosomes (> 0.99), thereby minimizing false homozygotes.

Given a heterozygous locus L with alleles A and B, the probability of sampling both alleles at the locus from a single individual is dependent on the sequencing depth for that locus. Let p_A be the probability of sampling allele A from locus L during the sequencing process, and p_B be the probability of sampling allele B from the same locus. Furthermore, let 'd' be the minimum number of reads required to confidently identify an allele (the arbitrary error-filtering coverage requirement), and 'n' the number of reads sampled at locus L. Then, for locus L, the probability of sampling only one of the alleles in a heterozygote at or above the coverage threshold ‘d’ is given by:

$$P(L)=\sum_{i=0}^{d-1} p_A^{n-i} * p_B^{i} + \sum_{j=0}^{d-1} p_B^{n-j} * p_A^{j}$$

Which can be further simplified as

$$P(L)=p_A^{n} * \sum_{i=0}^{d-1} \left(\frac{p_B}{p_A}\right)^{i} + p_B^{n} * \sum_{j=0}^{d-1} \left(\frac{p_A}{p_B}\right)^{j} \quad \text{(i)}$$

Assuming that for a heterozygous locus in a diploid organism both alleles are equally likely to be sampled, let $p_1 = p_A = p_B$ be the probability of sampling one of the alleles. Therefore, equation (i) becomes:

$$P(L)=2 * p_1^{n} * d \quad \text{(ii)}$$

and solving for n we have:

$$n=\left(\ln\left(p_1\right)\right)^{-1} * \ln\left(\frac{P(L)}{2*d}\right) \quad \text{(iii)}$$

The value P(L) above represents the binomial probability of falsely inferring a heterozygous locus to be homozygous due to read sampling error. Specifically, it represents the probability of having less than 'd' reads for at least one of the two alleles. Therefore, if a locus with 'n' reads assigned to it appears homozygous, it has a probability of P(L) of being a heterozygous locus erroneously assigned to the homozygous class due to read sampling alone, since it would have to have sampled at least n-d+1 copies of one allele while sampling d-1 or fewer of the other allele.

For a constant 'd', the P(L) value decreases as 'n' increases. Therefore, for any value of 'n' larger than a selected value, this probability is smaller than the probability for the selected value. Setting the value of P(L) to be 0.01, $p_1 = 0.5$, and requiring at least 4 identical reads in order to confidently call a haplotype, equation iii gives a required read depth at a locus of 10 reads (rounded to the nearest larger integer). Any value of 'n' >= 10 will have a probability of < 0.01 that at least one allele at a heterozygous locus is not sampled to sufficient depth to pass the 'd'=4 filter (which would result in a false homozygousity inference for the locus). In order to minimize false homozygosity due to sampling error we only analyzed clusters (loci) that had 11 or more reads assigned to them within the single individual.

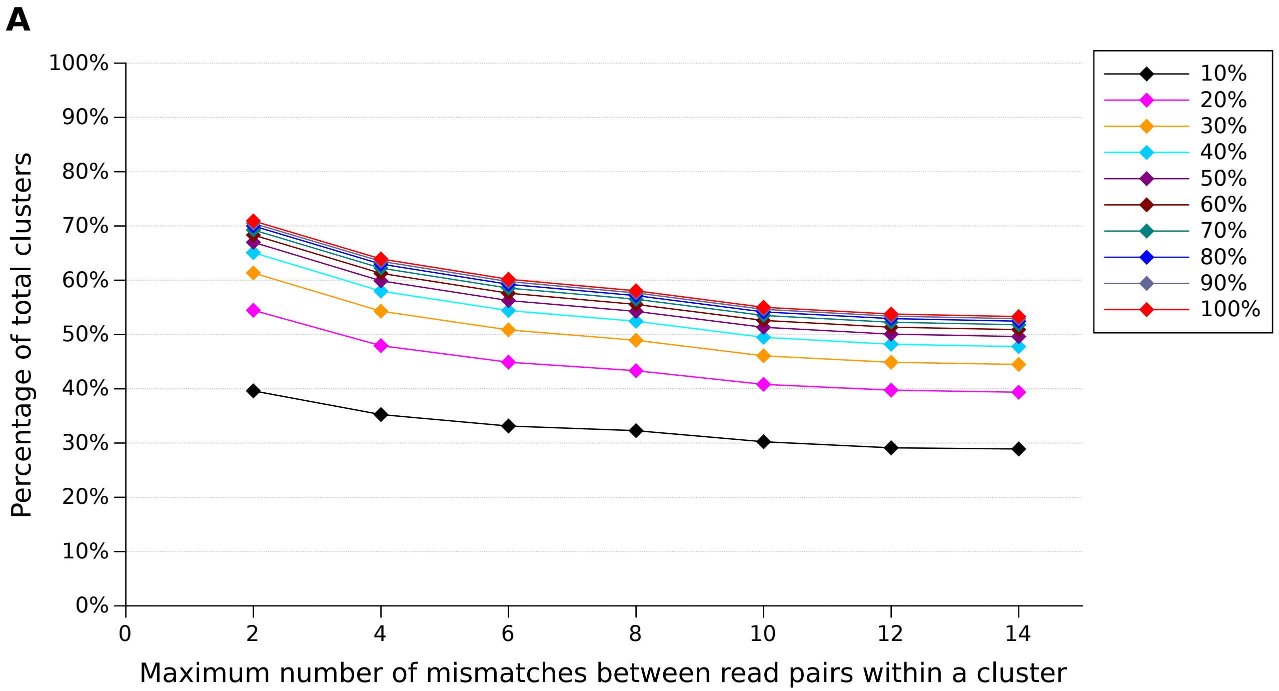
A
Percentage of total clusters
100%
90%
80%
70%
60%
50%
40%
30%
20%
10%
0%
0
2
4
6
8
10
12
14
Maximum number of mismatches between read pairs within a cluster
10%
20%
30%
40%
50%
60%
70%
80%
90%
100%

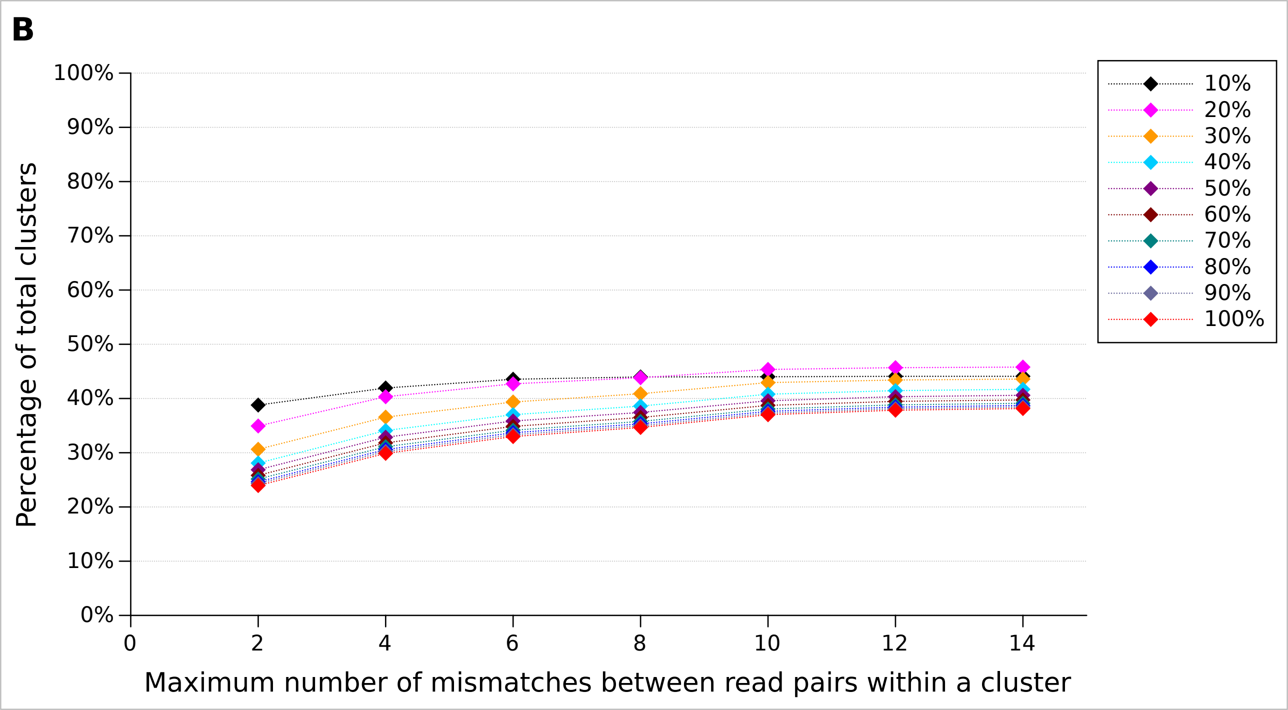

B
Percentage of total clusters
100%
90%
80%
70%
60%
50%
40%
30%
20%
10%
0%
0
2
4
6
8
10
12
14
Maximum number of mismatches between read pairs within a cluster
10%
20%
30%
40%
50%
60%
70%
80%
90%
100%

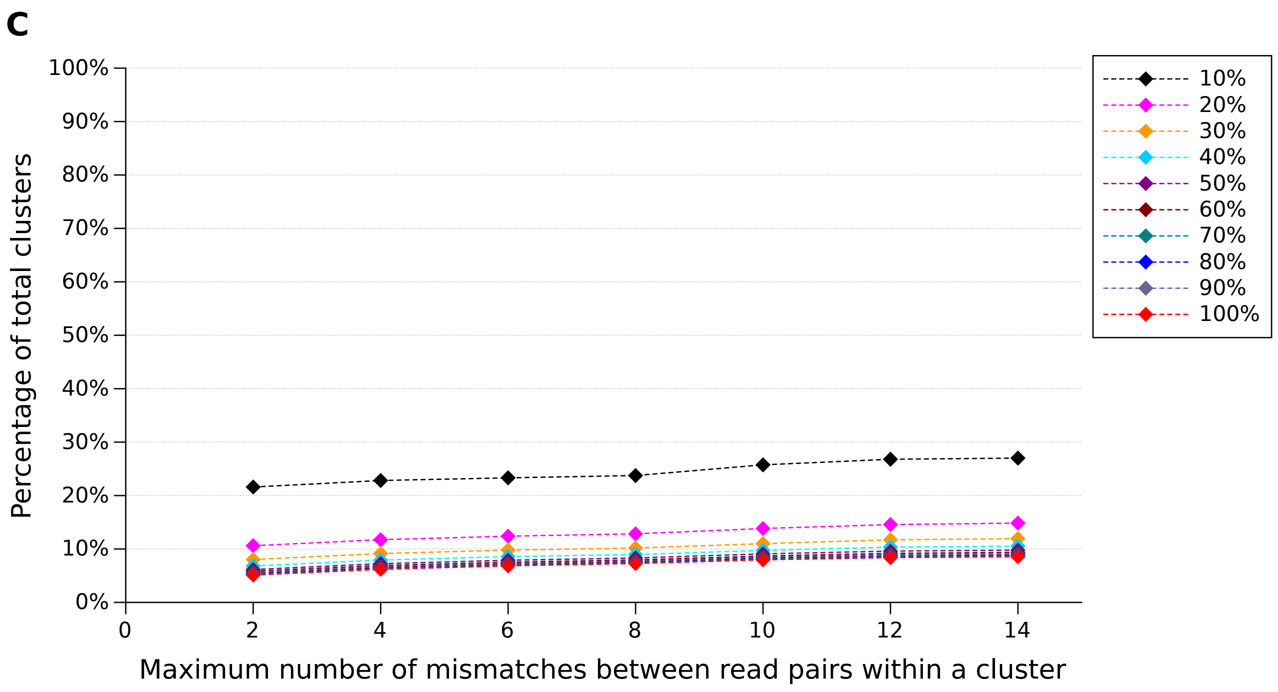
C
Percentage of total clusters
100%
90%
80%
70%
60%
50%
40%
30%
20%
10%
0%
0
2
4
6
8
10
12
14
Maximum number of mismatches between read pairs within a cluster
10%
20%
30%
40%
50%
60%
70%
80%
90%
100%

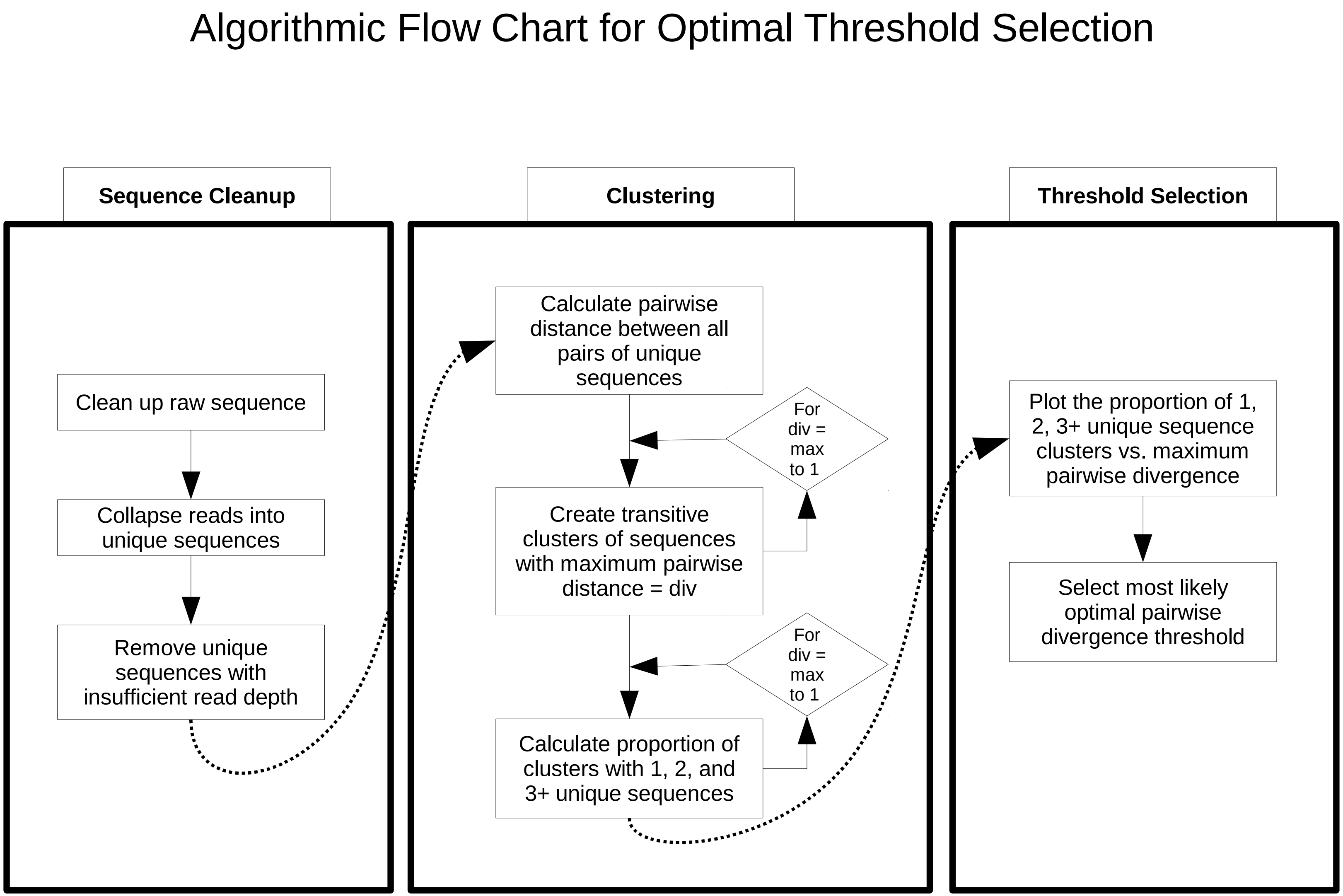
Algorithmic Flow Chart for Optimal Threshold Selection
Sequence Cleanup
Clustering
Threshold Selection
Clean up raw sequence
Collapse reads into unique sequences
Remove unique sequences with insufficient read depth
Calculate pairwise distance between all pairs of unique sequences
For div = max to 1
Create transitive clusters of sequences with maximum pairwise distance = div
For div = max to 1
Calculate proportion of clusters with 1, 2, and 3+ unique sequences
Plot the proportion of 1, 2, 3+ unique sequence clusters vs. maximum pairwise divergence
Select most likely optimal pairwise divergence threshold
